# Effect of Opioid-Sparing Anesthesia on Postoperative Nausea and Vomiting After Breast Surgery: A Single-Center Randomized Controlled Trial

**DOI:** 10.3390/jcm15124459

**Published:** 2026-06-09

**Authors:** Tae-Yun Sung, Youngjin Kim, Ji-Yoon Jung

**Affiliations:** 1 Department of Anesthesiology and Pain Medicine, Konyang University Hospital, Konyang University Myunggok Medical Research Institute, Konyang University College of Medicine, Daejeon 35365, Republic of Korea; unt1231@naver.com; 2 Department of General Surgery, Konyang University Hospital, Daejeon 35365, Republic of Korea; dotspot@naver.com

**Keywords:** opioid-sparing anesthesia, postoperative nausea and vomiting, breast surgery, dexmedetomidine, lidocaine, randomized controlled trial, perioperative care

## Abstract

**Background/Objectives**: Postoperative nausea and vomiting (PONV) remain frequent after breast surgery despite prophylaxis. This single-center, parallel-group randomized controlled trial evaluated whether intraoperative opioid-sparing anesthesia using dexmedetomidine and lidocaine reduced 48 h PONV compared with opioid-based anesthesia. **Methods**: Adult women undergoing elective breast surgery were randomized 1:1 to opioid-sparing anesthesia with dexmedetomidine and lidocaine or conventional opioid-based anesthesia with remifentanil. Participants and postoperative outcome assessors were blinded to group allocation; attending anesthesiologists were not blinded. All patients received standardized sevoflurane anesthesia, dexamethasone, ramosetron, quantitative neuromuscular monitoring, and postoperative fentanyl patient-controlled analgesia. The primary outcome was PONV within 48 h after surgery. **Results**: Among 68 randomized patients, 67 were analyzed (opioid-sparing group, *n* = 33; control group, *n* = 34). PONV within 48 h occurred in 8 patients (24.2%) in the opioid-sparing group and 28 patients (82.4%) in the control group (risk ratio, 0.29; 95% confidence interval, 0.16–0.55; *p* < 0.001; absolute risk reduction, 58.1%; number needed to treat, 1.7). Rescue antiemetic use was lower in the opioid-sparing group in the postanesthesia care unit and at 1 h postoperatively. Pain scores and cumulative opioid consumption were comparable. No Clavien–Dindo grade III or higher complications occurred. **Conclusions**: Intraoperative opioid-sparing anesthesia was associated with lower 48 h PONV after breast surgery without apparent compromise in analgesia. These single-center findings, from a trial registered after enrollment of one participant, require confirmation in larger prospectively registered multicenter trials. Trial Registration: Clinical Research Information Service (CRIS), KCT0009829. Registered on 10 October 2024.

## 1. Introduction

Postoperative nausea and vomiting (PONV) remains one of the most common and distressing complications after general anesthesia [1]. PONV is associated with delayed recovery, unanticipated hospital admission, increased healthcare utilization, and reduced patient satisfaction [2]. Patients undergoing breast surgery are predominantly female and are frequently exposed to volatile anesthetics and perioperative opioids, placing many in a high-risk category for PONV [2,3,4,5]. Although antiemetic prophylaxis reduces risk, PONV remains clinically relevant in this population.

From a perioperative clinical perspective, PONV is a modifiable outcome that can affect patient comfort, early recovery, and healthcare resource use. Strategies that reduce opioid-related adverse effects while preserving analgesia are therefore relevant to contemporary perioperative care pathways.

Intraoperative opioid administration is a modifiable contributor to PONV. Opioids promote nausea and vomiting through central effects on the chemoreceptor trigger zone and area postrema and through peripheral effects on gastrointestinal motility and gastric emptying [6,7]. These adverse effects have led to increasing interest in multimodal anesthetic strategies that reduce intraoperative opioid exposure while maintaining analgesic adequacy.

Opioid-sparing or opioid-free anesthesia has been evaluated in several surgical populations, including bariatric surgery, laparoscopic cholecystectomy, thoracic surgery, gynecologic surgery, and breast cancer surgery [8,9,10,11,12,13,14]. However, reported protocols and outcomes vary substantially, and many studies combine opioid avoidance with regional anesthesia or total intravenous anesthesia. Therefore, the incremental effect of a systemic intraoperative opioid-sparing regimen in patients undergoing breast surgery under volatile anesthesia and standardized antiemetic prophylaxis remains incompletely defined.

We conducted a prospective, single-center randomized controlled trial to evaluate whether an intraoperative opioid-sparing regimen using dexmedetomidine and lidocaine reduces the incidence of PONV within 48 h after breast surgery compared with opioid-based anesthesia. We hypothesized that intraoperative opioid sparing would reduce PONV without increasing postoperative pain or opioid consumption.

## 2. Materials and Methods

### 2.1. Study Design and Participants

This prospective, single-center, randomized controlled trial was conducted at Konyang University Hospital, Daejeon, Republic of Korea, between October 2024 and November 2025. The study protocol was approved by the Institutional Review Board of Konyang University Hospital on 29 July 2024 (approval no. KYUH 2024-07-006), and written informed consent was obtained from all participants before enrollment. The trial was conducted in accordance with the Declaration of Helsinki and reported according to the CONSORT 2025 statement.

The trial was registered with the Clinical Research Information Service (CRIS; registration no. KCT0009829) on 10 October 2024. At the time of trial registration, one participant had already been enrolled. No changes were made to the eligibility criteria, interventions, outcome definitions, assessment time points, or statistical analysis plan after trial registration, and no protocol deviations occurred after registration.

Eligible participants were women aged 19–75 years with American Society of Anesthesiologists physical status I–III who were scheduled for elective breast surgery under general anesthesia. Exclusion criteria were planned same-day discharge, expected postoperative mechanical ventilation, reoperation within 48 h after surgery, contraindications to any study medication, psychiatric illness or cognitive dysfunction, chronic opioid use, inability to provide informed consent, or refusal to participate. Baseline risk factors for PONV, including smoking status and a history of PONV or motion sickness, were recorded to calculate the simplified Apfel risk score for each patient [2].

### 2.2. Randomization and Blinding

Participants were randomly assigned in a 1:1 ratio to either the conventional opioid-based anesthesia group or the opioid-sparing anesthesia group. The randomization sequence was generated using R software, version 3.6.3 (R Foundation for Statistical Computing, Vienna, Austria) with a fixed seed, using simple randomization without blocking or stratification. Group assignments were concealed using sequentially numbered, opaque, sealed envelopes, which were opened immediately before induction of anesthesia.

Because of the nature of the intervention, the attending anesthesiologists could not be blinded. However, participants, postanesthesia care unit (PACU) nurses, and ward staff responsible for postoperative outcome assessment were blinded to group allocation.

### 2.3. Anesthesia and Perioperative Management

All participants received standard monitoring, including electrocardiography, noninvasive blood pressure monitoring, and pulse oximetry. Nitrous oxide was not used. Oxygen was administered via face mask before induction. Dexamethasone 5 mg was administered intravenously at induction for antiemetic prophylaxis, followed by propofol 1.5–2.0 mg/kg and rocuronium 0.6 mg/kg. Neuromuscular blockade was confirmed as a train-of-four count of zero using a quantitative neuromuscular monitoring device (TwitchView, Blink Device Company, Seattle, WA, USA). Endotracheal intubation was performed after loss of consciousness and adequate neuromuscular blockade. Depth of anesthesia was continuously monitored using a SedLine brain function monitor (Masimo Corp., Irvine, CA, USA).

In the conventional opioid-based anesthesia group, remifentanil was administered by target-controlled infusion starting at an effect-site concentration of 3 ng/mL and was titrated in increments of 0.5 ng/mL according to systolic blood pressure. Remifentanil was continued throughout surgery and discontinued at skin closure.

In the opioid-sparing anesthesia group, dexmedetomidine 1 μg/kg based on ideal body weight was infused over 10 min before induction, and lidocaine 1.5 mg/kg was administered intravenously. Dexmedetomidine and lidocaine infusions were then maintained at 0.5 μg/kg/h and 1.5 mg/kg/h, respectively, and were titrated according to hemodynamic responses. These doses were selected because they are commonly used in opioid-sparing multimodal anesthesia protocols and are within ranges reported to reduce sympathetic responses and opioid requirements while limiting hemodynamic adverse effects [8,15,16,17,18,19,20].

In both groups, anesthesia was maintained with sevoflurane adjusted to maintain a SedLine index of 25–50. Neuromuscular blockade was maintained with intermittent rocuronium boluses and reversed at the end of surgery with sugammadex according to quantitative neuromuscular monitoring. Ramosetron 0.3 mg was administered intravenously at the end of surgery.

All participants received intravenous patient-controlled analgesia (PCA) containing fentanyl at a total prescribed dose of 20 μg/kg. The PCA was programmed as a 2 mL demand bolus with a 10 min lockout interval and no basal infusion. Actual fentanyl consumption was calculated from recorded PCA use and the patient-specific fentanyl concentration.

During the first postoperative hour in the PACU, fentanyl 1 μg/kg was used as rescue analgesia, and dexamethasone 5 mg or metoclopramide 10 mg was used as rescue antiemetic therapy as needed. After PACU discharge, postoperative pain and PONV were managed on the ward according to a standardized institutional protocol until postoperative day 2. Nonopioid analgesics, including acetaminophen or nonsteroidal anti-inflammatory drugs when not contraindicated, were used as first-line rescue analgesics. Tramadol or intravenous fentanyl was reserved for inadequate pain control. Rescue antiemetics included metoclopramide or dexamethasone according to clinician assessment and institutional practice.

### 2.4. Outcomes

The primary outcome was the incidence of PONV within 48 h after surgery. PONV was assessed at four predefined time points: on arrival in the PACU, 1 h after PACU admission, postoperative day 1, and postoperative day 2. Nausea was recorded as patient-reported nausea, and vomiting was defined as any episode of retching or expulsion of gastric contents. Assessments were performed by PACU nurses and ward staff who were unaware of group allocation, using a standardized checklist. Rescue antiemetic therapy was administered when patients reported nausea, experienced retching or vomiting, or requested treatment for nausea. A formal numerical nausea severity scale was not used. Antiemetics were not administered preemptively after surgery in the absence of nausea, retching, vomiting, or patient request. These criteria were applied according to a standardized institutional checklist by PACU nurses and ward staff who were blinded to group allocation.

Secondary outcomes included rescue antiemetic use, cumulative antiemetic dose through postoperative day 2, cumulative intravenous PCA consumption through postoperative day 1 and postoperative day 2, additional postoperative analgesic requirements, postoperative pain scores assessed using the numeric rating scale through postoperative day 2, and complications classified as Clavien-Dindo grade III or higher. Minor adverse events were identified from the anesthetic record and postoperative chart when clinically documented or when active treatment was required.

### 2.5. Statistical Analysis

The primary and secondary outcomes were analyzed in all randomized participants for whom outcome data were available. Categorical variables were compared using the chi-square test or Fisher’s exact test, as appropriate. Continuous variables were compared using Student’s *t* test or the Mann–Whitney U test according to data distribution. For the primary outcome, effect estimates were expressed as risk ratios with 95% confidence intervals (CIs), along with absolute risk reduction and number needed to treat.

Because of the modest sample size and limited number of outcome events, adjusted analyses were considered exploratory. A parsimonious logistic regression model was used to estimate the association between group allocation and PONV after adjustment for the prespecified baseline PONV risk measure, the Apfel score. Additional covariates were not included in the primary adjusted model to reduce the risk of overfitting. A supplementary model including clinically relevant covariates was performed as a sensitivity analysis and is presented as exploratory. All analyses were performed using R software. A two-sided *p*-value < 0.05 was considered statistically significant.

#### Sample Size Calculation

The sample size was calculated based on an institutional PONV incidence of 70% after breast surgery. A 50% relative reduction was considered clinically meaningful. Using G*Power version 3.1, with a two-sided α of 0.05 and power of 80%, 31 participants were required per group. Assuming a dropout rate of 10%, 34 participants per group were enrolled, for a total sample size of 68.

## 3. Results

### 3.1. Patient Flow and Baseline Characteristics

**Figure 1:**
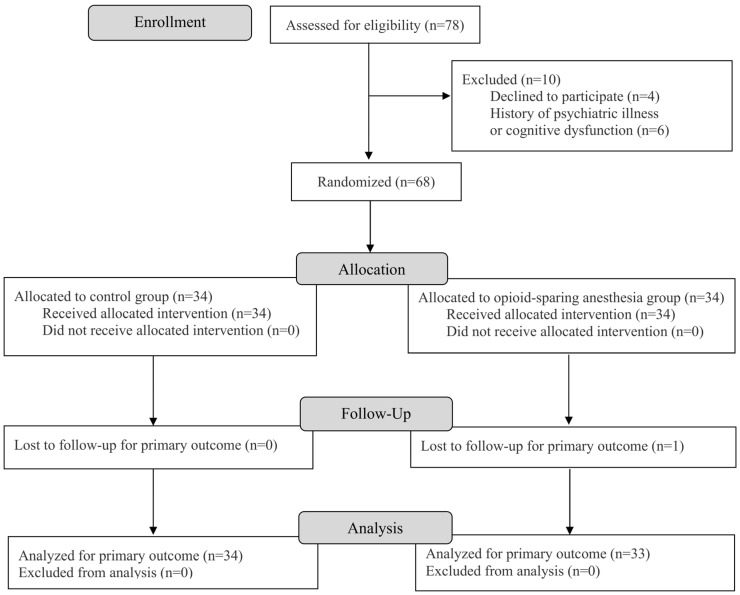
Flowchart of patient enrollment, exclusions, and allocation. A total of 78 patients were assessed for eligibility; 68 were randomized, and 67 were included in the final analysis (opioid-sparing anesthesia group, *n* = 33; control group, *n* = 34).

A total of 78 patients were assessed for eligibility. Six patients were excluded because of psychiatric illness or cognitive dysfunction, and four declined to participate. Consequently, 68 patients were randomized to either the opioid-sparing anesthesia group or the conventional opioid-based anesthesia group. One patient in the opioid-sparing group had incomplete 48 h follow-up data; therefore, outcome analyses were performed in 67 patients (opioid-sparing group, *n* = 33; control group, *n* = 34) (Figure 1).

Baseline demographic and clinical characteristics were comparable between groups (Table 1 and Supplementary Table S1). Intraoperative variables, including anesthesia duration, propofol dose, vasoactive agent use, estimated blood loss, and crystalloid administration, were also similar between groups (Supplementary Table S2).

### 3.2. Primary Outcome

**Figure 2:**
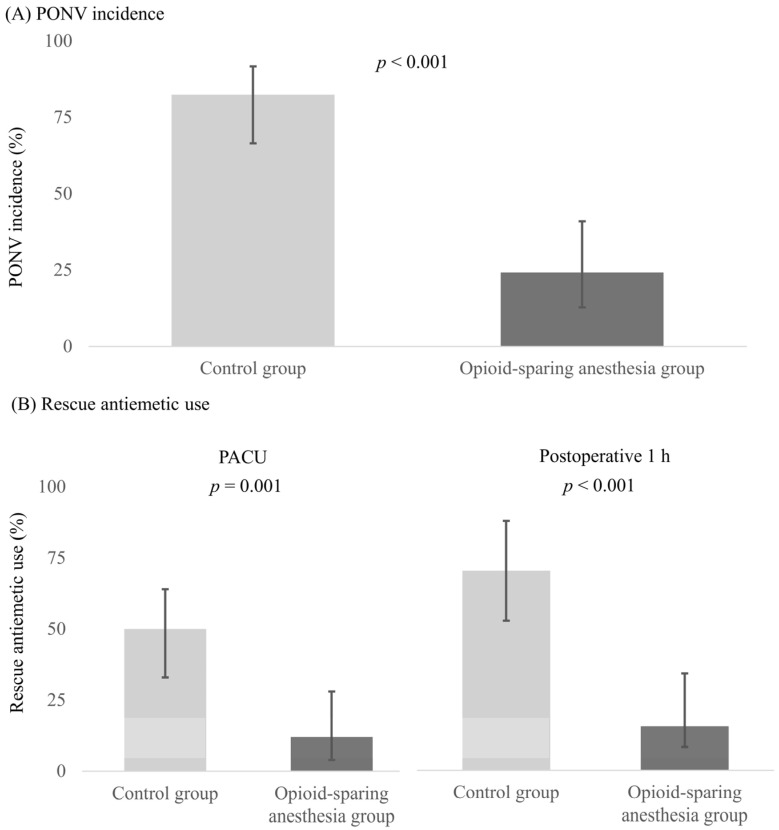
Incidence of postoperative nausea and vomiting (PONV) and rescue antiemetic use. (**A**) Incidence of PONV within 48 h after surgery. (**B**) Proportion of patients requiring rescue antiemetics in the postanesthesia care unit (PACU) and at 1 h postoperatively. Error bars represent 95% confidence intervals.

PONV within 48 h occurred in 8 patients (24.2%) in the opioid-sparing group and 28 patients (82.4%) in the control group (*p* < 0.001; Table 2 and Figure 2A). This corresponded to an absolute risk reduction of 58.1% (95% CI, 36–74%), a risk ratio of 0.29 (95% CI, 0.16–0.55), and an odds ratio of 0.07 (95% CI, 0.02–0.23). The number needed to treat was 1.7.

### 3.3. Secondary Outcomes and Adverse Events

Rescue antiemetic use was lower in the opioid-sparing group than in the control group in the PACU (12.1% vs. 50.0%, *p* = 0.001) and at 1 h postoperatively (15.2% vs. 67.6%, *p* < 0.001; Figure 2B). Postoperative pain scores did not differ significantly between groups at any time point. Cumulative opioid consumption over 48 h was numerically lower in the opioid-sparing group than in the control group (median [interquartile range], 288 [204–374] μg vs. 303.5 [232–407.5] μg), but the difference was not statistically significant (*p* = 0.22). Rescue analgesic requirements were similar between groups.

No Clavien–Dindo grade III or higher complications occurred in either group. Minor adverse events were rare and included one case of transient bradycardia in the opioid-sparing group and one case of transient hypertension in the control group.

### 3.4. Exploratory Adjusted Analysis

**Figure 3:**
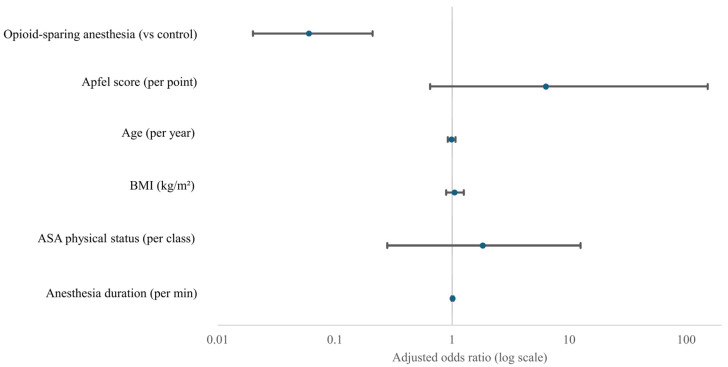
Exploratory adjusted predictors of postoperative nausea and vomiting (PONV). Logistic regression results are presented as adjusted odds ratios with 95% confidence intervals on a logarithmic scale. The vertical solid line indicates an odds ratio of 1.0.

In the parsimonious exploratory logistic regression model adjusted for Apfel score, opioid-sparing anesthesia remained associated with a lower risk of PONV (adjusted odds ratio, 0.07; 95% CI, 0.02–0.25; *p* < 0.001). A broader exploratory model including age, body mass index, American Society of Anesthesiologists physical status, and duration of anesthesia yielded similar point estimates but should be interpreted cautiously because of the limited number of events (Table 3). The broader exploratory model is visualized in Figure 3.

## 4. Discussion

In this single-center randomized trial, an intraoperative opioid-sparing regimen using dexmedetomidine and lidocaine was associated with a lower incidence of PONV within 48 h after breast surgery than opioid-based anesthesia. This reduction was observed despite standardized antiemetic prophylaxis and postoperative fentanyl PCA use in both groups. Postoperative pain scores, rescue analgesic use, and cumulative opioid consumption were not significantly different between groups.

For a broad clinical readership, the main relevance of these findings is not that the protocol represents a universally applicable opioid-free pathway, but that intraoperative anesthetic choices may meaningfully influence a patient-centered postoperative outcome in a population with high baseline PONV risk. The rationale for opioid-sparing strategies extends beyond PONV prevention. Although perioperative opioids remain essential for many patients, excessive or non-individualized opioid exposure may contribute to respiratory depression, sedation, ileus, urinary retention, pruritus, delayed recovery, opioid-induced hyperalgesia, and persistent postoperative opioid use. Contemporary perioperative care therefore emphasizes judicious, individualized opioid exposure within multimodal analgesia and enhanced recovery frameworks [8,15,21,22,23].

The magnitude of the observed effect was large and should be interpreted in the context of the study design and patient population. Breast surgery is a high-risk setting for PONV because most patients are female and frequently receive volatile anesthesia and opioids [2,3,4,5]. Most participants in the present trial had Apfel scores of 3 or 4. The control group incidence of 82.4% was higher than the approximate risk commonly predicted for an Apfel score of 3, but several factors may explain this observation. First, the primary outcome was cumulative 48 h PONV and included patient-reported nausea, which increases event capture compared with vomiting-only or PACU-only outcomes. Second, all patients received postoperative fentanyl PCA, which may have increased the baseline risk of PONV in both groups. Third, breast surgery patients are a relatively homogeneous high-risk population, so the Apfel score may have limited discrimination within this cohort [24].

The present findings are broadly consistent with previous reports suggesting that opioid-sparing or opioid-free strategies can reduce PONV or opioid-related adverse effects in selected surgical populations [8,9,10,11,12,13,14,17]. We acknowledge that the relationship between perioperative opioid exposure and PONV has been extensively investigated, and that the analgesic and opioid-sparing properties of dexmedetomidine and lidocaine have also been described in prior studies. Therefore, the contribution of the present study does not lie in demonstrating entirely novel pharmacologic effects of these agents. Rather, this trial provides procedure-specific randomized evidence regarding the application of a systemic intraoperative opioid-sparing regimen using dexmedetomidine and lidocaine in patients undergoing breast surgery under volatile anesthesia, standardized antiemetic prophylaxis, and postoperative fentanyl PCA. Our study does not establish that opioid-sparing anesthesia should be adopted routinely for all breast surgery patients. Rather, it suggests that, in patients undergoing breast surgery, who commonly have recognized risk factors for PONV, intraoperative opioid sparing with dexmedetomidine and lidocaine may be associated with a clinically important reduction in 48 h PONV.

The mechanisms underlying this association are likely multifactorial. Opioids can promote nausea and vomiting through central μ-opioid receptor effects in the area postrema and chemoreceptor trigger zone and through delayed gastric emptying and impaired gastrointestinal motility [6,7,16]. Remifentanil has an ultrashort context-sensitive half-time, but intraoperative exposure may still influence early postoperative nausea through opioid receptor activation, postoperative opioid sensitivity, and opioid-induced hyperalgesia or enhanced nociceptive signaling in some settings [7,24]. Dexmedetomidine may reduce sympathetic activation and opioid requirements and has been associated with reduced PONV in perioperative studies [17,18]. Intravenous lidocaine may improve postoperative recovery and gastrointestinal function and can reduce nociceptive input [19,20]. Therefore, the observed effect may reflect both avoidance of intraoperative remifentanil and pharmacologic effects of the nonopioid adjuncts.

An important feature of this study is that postoperative opioid exposure was not eliminated. Both groups received fentanyl-based PCA, and cumulative postoperative opioid consumption did not differ significantly. Therefore, the intervention should be understood as an intraoperative opioid-sparing anesthetic regimen rather than a fully opioid-free perioperative pathway. The substantial difference in PONV despite similar postoperative PCA use suggests that intraoperative opioid exposure may be particularly relevant for early PONV in this setting. However, this interpretation remains hypothesis-generating because the study was not designed to isolate the independent effects of remifentanil avoidance, dexmedetomidine, or lidocaine.

The adjusted analysis also requires caution. In the original model including multiple covariates, the direction and magnitude of the treatment effect were similar, although the number of outcome events was limited. For this reason, the adjusted analysis was revised to a parsimonious exploratory model adjusted only for the Apfel score, and the broader model is presented as a sensitivity analysis. The unadjusted risk ratio, absolute risk reduction, and number needed to treat remain the most transparent estimates of the primary outcome.

This study has several strengths. The randomized design reduces selection bias, and group allocation was concealed. Outcome assessment was performed by PACU and ward staff who were unaware of group allocation. The anesthetic protocol was standardized, neuromuscular monitoring was quantitative, nitrous oxide was not used, and both groups received the same antiemetic prophylaxis and postoperative PCA regimen. These features reduce several potential confounders relevant to PONV.

Several limitations should be acknowledged. First, this was a single-center study with a modest sample size, which limits generalizability and precision. Second, simple randomization without blocking or stratification may be suboptimal in small trials, although baseline characteristics were comparable between groups. Third, anesthesiologists could not be blinded, which may have introduced performance bias. Fourth, trial registration was completed after enrollment had begun because of an administrative delay; however, only one participant had been enrolled before registration. No changes were made to the eligibility criteria, interventions, outcome definitions, assessment time points, or statistical analysis plan after trial registration, and no protocol deviations occurred after registration. Fifth, all patients received postoperative fentanyl PCA; therefore, this study does not evaluate a fully opioid-free perioperative pathway. Sixth, the study population consisted exclusively of women undergoing breast surgery, limiting applicability to male patients or surgical populations with lower baseline PONV risk. Finally, we did not assess patient-centered recovery measures such as the quality of recovery score.

## 5. Conclusions

In this single-center randomized trial, intraoperative opioid-sparing anesthesia using dexmedetomidine and lidocaine was associated with a lower incidence of PONV within 48 h after breast surgery than opioid-based anesthesia, without apparent compromise in postoperative analgesia. Given the modest sample size, trial registration after enrollment of one participant, postoperative fentanyl use in both groups, and the single-center design, these findings should be interpreted cautiously and warrant confirmation in larger, prospectively registered multicenter trials before routine adoption.

## Figures and Tables

**Table 1:** Baseline demographic and preoperative characteristics.

Variable	Opioid-Sparing Anesthesia Group (*n* = 33)	Control Group (*n* = 34)	*p* Value
Age, years	56.1 ± 9.1	53.2 ± 11.1	0.23
Height, cm	158.4 [153.0–160.0]	158.5 [155.0–162.0]	0.72
Weight, kg	55.2 [48.1–63.4]	59.3 [55.2–63.6]	0.11
Body mass index, kg/m^2^	22.4 [19.4–25.4]	23.8 [21.4–25.4]	0.11
History of PONV	3 (9.1%)	3 (8.8%)	0.95
Apfel score			0.96
3	30 (90.9%)	31 (91.2%)	
4	3 (9.1%)	3 (8.8%)	
ASA physical status			0.81
I	0 (0.0%)	1 (2.9%)	
II	28 (84.8%)	28 (82.4%)	
III	5 (15.2%)	5 (14.7%)	
Hypertension	6 (18.2%)	9 (26.5%)	0.60
Diabetes mellitus	4 (12.1%)	2 (5.9%)	0.42
Diagnosis			0.44
Ductal carcinoma in situ	0 (0.0%)	3 (8.8%)	
Intraductal papilloma	1 (3.0%)	1 (2.9%)	
Malignant neoplasm of breast	31 (94.0%)	29 (85.4%)	
Microcalcification	1 (3.0%)	1 (2.9%)	
Surgery			0.62
Modified radical mastectomy	10 (30.3%)	15 (44.1%)	
Partial mastectomy	1 (3.0%)	0 (0.0%)	
Partial mastectomy with ALND	19 (57.6%)	16 (47.1%)	
Partial mastectomy with SLNB	2 (6.1%)	1 (2.9%)	
Total mastectomy	1 (3.0%)	2 (5.9%)	

Values are expressed as mean ± SD, median [interquartile range], or number of patients (%). ASA, American Society of Anesthesiologists; ALND, axillary lymph node dissection; PONV, postoperative nausea and vomiting; SLNB, sentinel lymph node biopsy.

**Table 2:** Primary and secondary postoperative outcomes.

Outcome	Opioid-Sparing Anesthesia Group (*n* = 33)	Control Group (*n* = 34)	*p* Value
Primary outcome			
PONV within 48 h	8 (24.2%)	28 (82.4%)	<0.001
Secondary outcomes			
Postoperative antiemetic use in PACU			0.001
None	29 (87.9%)	17 (50.0%)	
Dexamethasone	4 (12.1%)	15 (44.1%)	
Metoclopramide	0 (0.0%)	2 (5.9%)	
Postoperative antiemetic use at 1 h			<0.001
None	28 (84.8%)	11 (32.4%)	
Dexamethasone	3 (9.1%)	6 (17.6%)	
Metoclopramide	2 (6.1%)	17 (50.0%)	
Postoperative antiemetic use at 24 h			0.51
None	33 (100.0%)	32 (94.1%)	
Metoclopramide	0 (0.0%)	2 (5.9%)	
Postoperative pain score (NRS)			
PACU	2.0 [2.0–3.0]	3.0 [2.0–3.0]	0.13
1 h postoperatively	3.0 [2.0–3.0]	3.0 [3.0–3.0]	0.32
24 h postoperatively	3.0 [2.0–3.0]	3.0 [2.0–3.0]	0.93
48 h postoperatively	2.0 [2.0–3.0]	2.0 [2.0–3.0]	0.72
Maximum NRS score	3.0 [3.0–4.0]	3.0 [3.0–4.0]	0.34
Postoperative PCA consumption, mL			
1 h postoperatively	32.0 [30.0–36.0]	36.0 [33.0–40.0]	0.09
1–24 h postoperatively	150.0 [108.0–204.0]	150.0 [130.0–255.0]	0.11
24–48 h postoperatively	78.0 [55.0–144.0]	84.0 [70.0–120.0]	0.62
Postoperative analgesic use in PACU			0.95
None	31 (93.9%)	32 (94.1%)	
Nonopioid	2 (6.1%)	2 (5.9%)	
Postoperative analgesic use at 1 h			0.94
None	31 (93.9%)	30 (88.3%)	
Nonopioid	2 (6.1%)	3 (8.8%)	
Opioid	0 (0.0%)	1 (2.9%)	
Postoperative analgesic use at 24 h			0.72
None	23 (69.7%)	20 (58.8%)	
Nonopioid	2 (6.1%)	2 (5.9%)	
Opioid	8 (24.2%)	12 (35.3%)	
Postoperative analgesic use at 48 h			0.95
None	32 (97.0%)	33 (97.1%)	
Nonopioid	0 (0.0%)	1 (2.9%)	
Opioid	1 (3.0%)	0 (0.0%)	
Cumulative opioid consumption over 48 h, fentanyl equivalents (μg)	288.0 [204.0–374.0]	303.5 [232.0–407.5]	0.22

Values are expressed as median [interquartile range] or number of patients (%). NRS, numeric rating scale; PACU, postanesthesia care unit; PCA, patient-controlled analgesia; PONV, postoperative nausea and vomiting. No statistical comparison was performed when there was no variability between groups.

**Table 3:** Exploratory logistic regression analysis for postoperative nausea and vomiting.

Model and Variable	Odds Ratio	95% CI	*p* Value
Parsimonious exploratory model			
Opioid-sparing anesthesia vs. control	0.07	0.02–0.25	<0.001
Apfel score, per point	6.00	0.65–142	0.13
Broader sensitivity model			
Opioid-sparing anesthesia vs. control	0.07	0.02–0.23	<0.001
Apfel score, per point	6.32	0.65–152	0.15
Age, per year	0.99	0.92–1.07	0.84
Body mass index, kg/m^2^	1.05	0.89–1.26	0.55
ASA physical status, per class	1.83	0.28–12.5	0.52
Duration of anesthesia, per min	1.01	0.99–1.03	0.40

The parsimonious model was prespecified for the revised analysis to reduce overfitting and included group allocation and the Apfel score. The broader model is presented only as a sensitivity analysis and should be interpreted cautiously because of the limited number of outcome events. ASA, American Society of Anesthesiologists; CI, confidence interval.

## Data Availability

The datasets generated and/or analyzed during the current study are not publicly available because they contain potentially identifiable clinical information but are available from the corresponding author upon reasonable request and with appropriate ethical approval.

## Supplementary Materials

The following supporting information can be downloaded at https://www.mdpi.com/article/10.3390/jcm15124459/s1: Table S1: Detailed underlying diseases and medication use; Table S2: Intraoperative characteristics.

## Abbreviations

The following abbreviations are used in this manuscript:

ALND	Axillary lymph node dissection
ASA	American Society of Anesthesiologists
CI	Confidence interval
CRIS	Clinical Research Information Service
NRS	Numeric rating scale
PACU	Postanesthesia care unit
PCA	Patient-controlled analgesia
PONV	Postoperative nausea and vomiting
RCT	Randomized controlled trial
SLNB	Sentinel lymph node biopsy
