# Supplementary material for: Effect of Opioid-Sparing Anesthesia on Postoperative Nausea and Vomiting After Breast Surgery: A Single-Center Randomized Controlled Trial
Source: J Clin Med. 2026 Jun 9;15(12):4459. doi: 10.3390/jcm15124459 (PMC13301233; doi:10.3390/jcm15124459)
Supplement: Supplementary file 1 [file jcm-15-04459-s001.zip › Table S1. Detailed underlying diseases and medication use.pdf]

Table S1. Detailed underlying diseases and medication use

	Control group (n = 34)	Opioid-sparing anesthesia group (n = 33)	P value
**Underlying diseases, n (%)**			
Thyroid disease	2 (5.9%)	2 (6.1%)	0.95
Liver disease	1 (2.9%)	1 (3.0%)	0.95
Kidney disease	2 (5.9%)	2 (6.1%)	0.95
Respiratory disease	0 (0.0%)	3 (9.1%)	0.11
Cardiovascular disease	0 (0.0%)	1 (3.0%)	0.52
Neurological disease	3 (8.8%)	2 (6.1%)	0.94
Endocrine or rheumatologic disease	1 (2.9%)	1 (3.0%)	0.95
Cerebrovascular disease	1 (2.9%)	0 (0.0%)	0.94
**Medications, n (%)**			
Renin-angiotensin system inhibitors	6 (17.6%)	3 (9.1%)	0.52
Calcium channel blockers	7 (20.6%)	4 (12.1%)	0.54
Beta-blockers	1 (2.9%)	1 (3.0%)	0.95
Oral hypoglycemic agents	2 (5.9%)	3 (9.1%)	0.71
Insulin	0 (0.0%)	1 (3.0%)	0.52
Aspirin	0 (0.0%)	1 (3.0%)	0.52
**Others, n (%)**			
History of surgeries	9 (26.5%)	7 (21.2%)	0.82
Social drinking status	10 (29.4%)	11 (33.3%)	0.83

Values are expressed as number of patients (%).
