# Supplementary material for: Effect of Opioid-Sparing Anesthesia on Postoperative Nausea and Vomiting After Breast Surgery: A Single-Center Randomized Controlled Trial
Source: J Clin Med. 2026 Jun 9;15(12):4459. doi: 10.3390/jcm15124459 (PMC13301233; doi:10.3390/jcm15124459)
Supplement: Supplementary file 1 [file jcm-15-04459-s001.zip › Table S2. Intraoperative characteristics.pdf]

Table S2. Intraoperative characteristics

	**Control group (n = 34)**	**Opioid-sparing anesthesia group (n = 33)**	**P value**
Duration of anesthesia, min	115 [100-135]	100 [90-125]	0.21
Total propofol dose, mg	120 [100-120]	100 [100-120]	0.13
Total remifentanil dose, µg	304 [235-420]	-	
Total dexmedetomidine dose, µg	-	115 [90-135]	
Total lidocaine dose, mg	-	115 [97-135]	
Estimated blood loss, ml	100 [50-150]	100 [50-150]	0.62
Crystalloid administration, ml	400 [250-500]	300 [300-400]	0.53
Ephedrine use, n (%)	19 (55.9%)	22 (66.7%)	0.34
Nicardipine use, n (%)	5 (14.7%)	3 (9.1%)	0.71
Esmolol use, n (%)	2 (5.9%)	0 (0.0%)	0.52

Values are expressed as median [IQR] or number of patients (%). Remifentanil was administered only in the control group, whereas dexmedetomidine and lidocaine were administered only in the opioid-sparing anesthesia group; therefore, no between-group statistical comparison was performed.
